# Supplementary material for: WordCommentsAnalyzer: A windows software tool for qualitative research
Source: F1000Res. 2018 Sep 4;7:536. Originally published 2018 May 3. [Version 2] doi: 10.12688/f1000research.14819.2 (PMC6137411; doi:10.12688/f1000research.14819.2)
Supplement: Supplementary file 3 [file f1000research-7-17614-s0002.tgz › b2a79496-4ada-47c4-9682-a99ce867836d.zip!/Eight Iranian Health Journals/Yafteh _30.docx]

© Yafteh  . This work is licensed under CC BY 4.0 (https://creativecommons.org/licenses/by/4.0/).

Volume & Issue: دوره 13 - شماره 2

Title: تکثیر و تیپ‌بندی فلاژلین سودوموناس آئروژینوزا به روش مولکولی

http://yafte.lums.ac.ir/article-1-535-fa.html

بحث و نتیجه‌گیری: این روش می‌تواند در تشخیص سویه‌های فلاژل دار (متحرک) و فاقد فلاژل سودوموناس آئروژینوزا، تکثیر کامل توالی ژن fliC به منظورکلون‌سازی و بیان پروتئین فلاژلین نوترکیب کاربرد داشته و همچنین جایگزین روش تیپ بندی سرولوژیک فلاژل باشد. مواد و روش‌ها: پس از استخراج DNA ژنومی از دو سویه استاندارد M 8821 (فلاژلین تیپ a) و PAO1 (فلاژلین تیپ b) و چند سویه بالینی سودوموناس آئروژینوزا، با طراحی یک جفت پرایمر اختصاصی و واکنش زنجیره‌ای پلی‌مراز (PCR)، ژن کامل fliC سویه‌های مورد آزمایش تکثیر و از نظر طول قطعه ژنی مقایسه گردید. یافته‌ها: نتایج PCR و تعیین توالی نشان داد که طول ژن fliCدر سویه‌های تیپ a و b متفاوت و به ترتیب bp 1160 و bp 1460 می‌باشد. سودوموناس آئروژینوزا دارای یک فلاژل قطبی است که از زیرواحد‌های فلاژلین که توسط ژن fliC کد می‌گردد ساخته می‌شود. فلاژلین سویه‌های مختلف سودوموناس آئروژینوزا بر اساس واکنش با آنتی‌بادی‌های پلی‌کلونال و وزن مولکولی به دو تیپ سرولوژیک a یا b تقسیم می‌شود.هدف از این مطالعه تکثیر و تیپ‌بندی فلاژلین سودوموناس آئروژینوزا به روش مولکولی بود.

Title: اثر تجویز اسانس گیاه مرزه خوزستانی بر شدت آسیب کلیوی ناشی از تجویز سیس‌پلاتین در موش‌های صحرایی

http://yafte.lums.ac.ir/article-1-534-fa.html

سیس‌پلاتین از داروهای اصلی شیمی‌درمانی انواعی از سرطان است و نفروپاتی مهمترین عارضه آن محسوب می‌شود. رادیکالهای آزاد اکسیژن نقش مهمی در ایجاد سمیت کلیوی ناشی از این دارو دارند. برخی اسانسهای گیاهی حاوی ترکیبات آنتی‌اکسیدان هستند. هدف از این تحقیق تعیین اثر اسانس گیاه مرزه خوزستانی بر نفروپاتی ناشی از سیس‌پلاتین است. بحث و نتیجه‌گیری: تجویز همزمان اسانس مرزه خوزستانی با سیس‌پلاتین سمیت کلیوی آنرا کم نمی‌کند و ممکن است در دوزهای بالاتر نفروپاتی ناشی از سیس‌پلاتین را شدیدتر کند. یافته‌ها: تجویز اسانس با دوز mg/kg200 همزمان با سیس‌پلاتین منجر به مرگ حیوانات شد. تزریق اسانس به مقدار mg/kg100 نفروپاتی ناشی از سیس‌پلاتین را تشدید کرد و در دوز mg/kg50 شدت نفروپاتی تفاوت معنی‌داری با گروه کنترل نداشت. تزریق اسانس به‌تنهایی اثری بر عملکرد کلیه نداشت. مواد و روش‌ها: موشهای صحرایی نر ویستار 300-200 گرمی مورد استفاده قرار گرفتند. در گروه کنترل (10n=) سیس‌پلاتین (mg/kg5) و در گروههای مورد (در هر گروه 6n=) اسانس با دوزهای mg/kg50، 100 و 200 و سپس سیس‌پلاتین تزریق شد. در تعدادی از گروهها (در هر گروه 9-6n=) نیز اسانس و سپس نرمال سالین (بجای سیس‌پلاتین) تزریق شد. 3 روز بعد از تجویز سیس‌پلاتین (یا نرمال سالین) غلظت کراتینین و اوره پلاسما و کلیرانس کراتینین و کسر دفع سدیم بعنوان شاخصهای عملکرد کلیه سنجیده شد.

Title: بهبود بقا بیماران مبتلا به لوسمی لنفوبلاستیک حاد فاقد HLA-A3/11 برای KIR3DL2 دهنده پس از پیوند سلول های بنیادی خونساز از خواهر یا برادر با HLA مشابه بدون تخلیه سلول T

http://yafte.lums.ac.ir/article-1-438-fa.html

یک عامل بالقوه تاثیر گذار بر نتیجه پیوند سلول های بنیادی خونساز (HSCT) حضور سلول های کشنده طبیعی NK)) آلوری اکتیو مشتق از دهنده است. در این آنالیز گذشته نگر می‌توان تأثیر آلوری اکتیویتی NK بر اساس فقدان لیگاند KIR را بر روی بیماران مبتلا به لوسمی میلوئیدی حاد (AML) یا لوسمی لنفوبلاستیک حاد (ALL) تحت HSCT از خواهر یا برادر با HLA مشابه بدون تخلیه سلول T مطالعه نمود. بحث و نتیجه‌گیری: این نتایج نشان می دهد که فقدان لیگاند HLA کلاس یک در گیرنده برای KIR مهاری دهنده می تواند یک فاکتور پیشگویی کننده برای نتایج پیوند در پیوند سلول های بنیادی خونساز از خواهر یا برادر با HLA مشابه بدون تخلیه سلول T باشد و این که فقدان HLA-A3/11 برای KIR3DL2 دهنده ممکن است به بهبود بقا در بیماران مبتلا به ALL کمک نماید. یافته‌ها: فقدان لیگاند KIR بدون HLA-A3/11 تأثیری بر روی بقا بدون بیماری (DFS) ، بقا کلی (OS) یا عود در بیماران دریافت کننده پیوند به دلیل AML یا ALL نداشت. بااین وجود، فقدان لیگاند KIR با HLA-A3/11 به طور معنی داری بر DFS (04/0P=) و OS (02/0P=) موثر بود. مواد و روش‌ها: در کل 78 بیمار، شامل 40 بیمار مبتلا به AML و 38 بیمار مبتلا به ALL مطالعه شدند. تقریباً تمام بیماران یک رژیم یکسان آماده سازی ریشه کن کننده مغزاستخوان و پیشگیری کننده GVHD را دریافت کرده بودند. تمام افراد برای ژن های KIR و لیگاندهای HLA به روش واکنش زنجیره ای پلیمراز با استفاده از پرایمرهای اختصاصی توالی (PCR-SSP) تعیین ژنوتیپ گردیدند.

Title: بررسی سبک زندگی بیماران مبتلا به مولتیپل اسکلروزیس مراجعه کننده به انجمن ام.اس شهر تهران سال1387

http://yafte.lums.ac.ir/article-1-439-fa.html

بیماری مولتیپل اسکلروزیس غیر قابل پیش بینی و یکی از مهمترین بیماری های تغییر دهنده زندگی فرد است و بتدریج او را به سمت ناتوانی پیش می برد. بین سلامتی و سبک زندگی افراد ارتباط نزدیکی وجود دارد با اصلاح سبک زندگی می توان از بروز حملات بیماری و ناتوانی پیشرونده جلوگیری کرد لذا شناخت سبک زندگی جهت برنامه ریزی های آموزشی اهمیت دارد. این مطالعه با هدف بررسی و تعیین سبک زندگی بیماران مبتلا به مولتیپل اسکلروزیس مراجعه کننده به انجمن ام.اس شهر تهران سال 1387 صورت گرفته است بحث و نتیجه‌گیری: سبک زندگی واحد های مورد پژ‍وهش در برخی از ابعاد نامطلوب بود و این رفتارهای غیر بهداشتی می‌تواند بر شدت و عود بیماری و در نهایت کیفیت زندگی بیماران تأثیر گذار باشد. لذا تغییر و تعدیل و اصلاح سبک زندگی بیماران مبتلا به ام. اس ضروری به نظر می رسد که با برنامه های آموزشی جامع می توان به این مهم دست یافت. یافته‌ها: سبک زندگی 5/38 درصد واحد های مورد پژوهش در بعد مراقبت از خود مطلوب، 78 درصد نمونه ها در بعد تغذیه نسبتاً مطلوب و 5/61 درصد در بعد فعالیت جسمانی و ورزش نامطلوب بوده اند، 83 درصد از نظر عدم استعمال دخانیات مطلوب بوده اند، 45 درصد واحد های مورد پژوهش در زمینه الگوی خواب و استراحت و 5/50 درصد از نظر سازگاری با استرس نامطلوب بوده اند. یافته ها نشان داد که بین سبک زندگی در بعد مراقبت از خود با سطح تحصیلات مددجو (001/0>p)، داشتن منابع حمایتی(031/0= p) و دفعات عود بیماری (017/0= p) واحد های مورد پژوهش ارتباط آماری معنی داری وجود داشت. همچنین بین سبک زندگی در بعد تغذیه با وضعیت تأهل(015/0=p) و درآمد خانواده(001/0>p) نمونه های پژوهش ارتباط آماری معنی داری دیده شد. مواد و روش‌ها: این پژوهش یک مطالعه توصیفی- مقطعی است. تعداد 200 نفر از بیماران مبتلا به ام.اس مراجعه کننده به انجمن ام. اس ایران در سال 1387 که شرایط ورود به مطالعه را داشتند به عنوان نمونه انتخاب گردیدند نمونه گیری به صورت آسان و در دسترس بود. ابزار گردآوری داده ها پرسشنامه و روش جمع آوری آن مصاحبه بود . اعتبار ابزار از طریق اعتبار محتوی و اعتماد علمی آن به روش آزمون مجدد انجام گردید. در پایان داده های پژوهش با استفاده از آمار توصیفی و تحلیلی (کای دو، آزمون دقیق فیشر) و نرم افزار SPSS مورد تجزیه و تحلیل قرار گرفت.

Title: مطالعه ایزوترم و کینتیک جذب کروم شش ظرفیتی بر روی دانه های آلژینات کلسیم در محلولهای آبی

http://yafte.lums.ac.ir/article-1-440-fa.html

یکی از مهمترین معضلات زیست محیطی آلودگی آب و خاک به فلزات سنگین است. در این راستا هدف از این مطالعه حذف زیستی کروم (VI) از محلولهای آبی با استفاده از آلژینات کلسیم و تعیین مدلهای ایزوترم و کینتیک جذب بود. مواد و روش‌ها: با استفاده از راکتور جریان ناپیوسته، ظرفیت جذب زیستی آلژینات کلسیم جهت جذب کروم (VI) تحت تأثیر pH ، زمان اختلاط و غلظت فلز تعیین گردید. جهت تعیین باندهای فعال در جذب و تعیین خصوصیات جاذب از آنالیز اسپکتروفوتومتر مادون قرمز و میکروسکوپ الکترونی استفاده شد. بحث و نتیجه‌گیری: با توجه به مدل دوبنین رادوشویچ می توان ابراز داشت که فرآیند جذب کروم (VI) بر روی آلژینات کلسیم مکانیسمی شیمیایی دارد. همچنین با توجه به ماکزیمم ظرفیت جذب بدست آمده می توان ابراز داشت که آلژینات کلسیم جهت جذب زیستی کروم کارآمد و مؤثر است. یافته‌ها: با توجه به آنالیز اسپکتروفوتومتر مادون قرمز نشان داده شد که باندهای کربوکسیل و هیدروکسیل می توانند مهمترین نقش را در جذب کروم (VI) بر روی آلژینات کلسیم داشته باشند. pH بهینه 4 جهت جذب کروم (VI) بر روی آلژینات کلسیم بدست آمد. تطابق مطلوبی بین داده های آزمایشگاهی و ایزوترمهای مورد مطالعه بدست آمد (9/0R2>). ماکزیمم ظرفیت آلژینات کلسیم جهت جذب زیستی کروم(VI) 75/84 میلیگرم بر گرم بدست آمد. متوسط انرژی آزاد جذب کروم (VI) بر روی آلژینات کلسیم(E) 129/9 کیلوژول بر مول بدست آمد. علاوه بر آن، مدل کینتیکی درجه دوم کاذب نسبت به مدل درجه دوم کاذب جهت تطبیق داده های آزمایشگاهی مناسب‌تر گزارش شد (R2>0.97) و در طی 120 دقیقه تعادل جذب حاصل گردید.

Title: بررسی و مقایسه دمپایی s‏plint طراحی شده با اسپلینت موجود در بازار در درمان بیماری شصت کج (Hallux Valgus)

http://yafte.lums.ac.ir/article-1-441-fa.html

بیماری هالوکس والگوس یا انحراف شصت پا به سمت خارج،یک بیماری پیچیده است که در صورت عدم درمان باعث انحراف در سایر انگشتان پا می شود.این بیماری در خانم ها سه برابر شایع تر از آقایان است که بیمار ممکن است به علت تغییر شکل ناخوشایند پا ،اشکال در پوشیدن کفش های پنجه باریک و نامناسب و یا درد بر روی برجستگی قسمت داخلی مفصل به پزشک مراجعه کند . این بیماری ممکن است با چکشی شدن انگشت دوم توأم باشد. بحث و نتیجه گیری: این مطالعه نشان داد که با وجود اختلاف نظرهای زیادی که در درمان غیر جراحی هالوکس والگوس وجود دارد در صورتی که انحراف شصت پای بیماران بیش از حد نباشد به‌طوری که درمان جراحی نیاز نبود و می‌توان از این اسپلینت جهت درمان غیر جراحی استفاده کرد. یافته ها: انحراف شصت پای در بیمارانی که از دمپایی اسپلینت بطور مرتب استفاده کرده بودند بهبود قابل توجهی نسبت به بیمارانی که از اسپلینت موجود در بازار استفاده کردند،داشته و از نظر آماری هم اختلاف معنی داری بین دو گروه (001/0>p) وجود داشت. مواد و روش ها: این مطالعه یک مطالعه کوهورت می باشد که از بین بیماران مراجعه کننده به کلینیک ارتوپدی بیمارستان شهدای عشایر شهرستان خرم آباد باشکایت هالوکس والگوس و انحراف شصت به طور تصادفی، دو گروه 30 نفری انتخاب شدند. به یک گروه اسپلینت طراحی شده (دمپایی اسپلینت) و به گروه دیگر اسپلینت موجود در بازار (Night splint و پد لاانگشتی ) داده شد.بیماران در طی یک‌سال هر سه ماه مورد بررسی قرار گرفتند و در هر بار مراجعه با گرفتن عکس رادیوگرافی رخ کف پا در حالت ایستاده ، زوایا اندازه گیری شد. پس از وارد کردن داده ها در نرم افزار spss نتایج با استفاده از آزمون اندازه گیری با تکرار ((Repeated measure تجزیه و تحلیل گردید.

Title: اثرات آنتی اکسیدانی عصاره آبی میوه عناب بر استرس اکسیداتیو ناشی از اتانول در کبد و کلیه موش‌های صحرایی نر

http://yafte.lums.ac.ir/article-1-442-fa.html

به خوبی می‌دانیم که بیماریزائی اتانول در کبد و کلیه‌ها در ارتباط مستقیم با افزایش رادیکال‌های آزاد و استرس اکسیداتیو است که منجر به اختلالات ساختمانی و عملکردی در این دو ارگان حیاتی می‌شود. مواد و روش‌ها: 56 عدد موش بزرگ آزمایشگاهی نر از نژاد ویستار در 8 گروه قرار گرفته و به ترتیب زیر درمان شدند: گروه کنترل(سالین نرمال)،گروه اتانول (اتانول 4 گرم بر کیلوگرم وزن بدن)، 3 گروه سه دوز از عصاره میوه عناب (50، 100 و 200 میلی‌گرم بر کیلو گرم وزن بدن)، و3 گروه سه دوز از عصاره میوه عناب و اتانول(با همان سه دوز در کنار اتانول 4 گرم بر کیلوگرم وزن بدن). همه درمان‌ها روزی یک مرتبه بصورت داخل معده‌ای به مدت 60 روز پیاپی صورت گرفت. نمونه‌های سرمی و بافت کبد و کلیه جهت ارزیابی ترکیبات سرمی، فعالیت آنزیم‌های آنتی‌اکسیدان بافتی، و نیز میزان مواد واکنش دهنده با تیوباربیتوریک اسید از موش‌ها اخذ شد. بحث و نتیجه‌گیری: عصاره آبی میوه عناب بعنوان پیش درمان، کبد و کلیه را در برابر آسیب اکسیداتیو ناشی از اتانول محفاظت می‌نماید. یافته‌ها: فعالیت کبدی آنزیم‌های سوپر اکسید دیسموتاز و گلوتاتیون پراکسیداز(GPx)در گروه اتانول، در مقایسه با گروه کنترل به طور معنی‌داری کاهش یافت، در حالیکه عصاره میوه عناب(200 میلی‌گرم) توانست تنها فعالیت گلوتاتیون پراکسیداز را بطور معنی‌دار افزایش دهد. فعالیت GPxکلیوی در مصرف اتانول به طور معنی‌داری نسبت به گروه کنترل افزایش نشان داد. همچنین فعالیت GPxدر گروه عصاره میوه عناب و الکل(200 میلی گرم و 4 گرم) نسبت به گروه اتانول کاهش معنی‌دار نشان داد. در مطالعه حاضر میزان TBARS که نشان‌دهنده پروکسیداسیون چربی است در بافت کبد گروه اتانول،در مقایسه با گروه کنترل افزایش معنی‌دار نشان داد(05/0 p <).

Title: بررسی فراوانی انواع توده های دیواره قفسه صدری جراحی شده و سرانجام درمان آنها در بیمارستان الزهرا(س) اصفهان در سال‌های 1386-1378

http://yafte.lums.ac.ir/article-1-443-fa.html

توده‌های دیواره‌ قفسه‌ صدری طیف وسیعی از ضایعات خوش‌خیم و بدخیم هستند. در مطالعات مختلف نتایج متفاوتی پیرامون ضایعات قفسه‌ صدری گزارش شده است ولی هنوز اطلاعات دقیقی از انواع این ضایعات در بیماران در دست نیست. هدف از این مطالعه تعیین فراوانی انواع توده‌های دیواره‌ قفسه‌‌ صدری درکل و بر اساس متغیرهای اپیدمیولوژیک و چگونگی پاسخ به درمان و همچنین بررسی سرانجام این بیماران است. مواد و روش‌ها: این مطالعه‌ توصیفی تحلیلی در سال 1388 در مرکز آموزشی درمانی الزهرا(س) اصفهان به انجام رسید. جامعه آماری مورد مطالعه شامل تمامی بیمارانی بود که از ابتدای سال 1378 تا پایان سال 1386 با تشخیص تومور جدار قفسه صدری در این بیمارستان تحت عمل قرار گرفته بودند. روش نمونه گیری در این مطالعه به صورت سرشماری بود و طی آن تعداد 112 مورد عمل جراحی که طی مدت زمان مذکور انجام گرفته بود مورد بررسی قرار گرفت. بحث و نتیجه‌گیری: طبق نتایج به دست آمده از این مطالعه حدود 59 درصد تومور های دیواره قفسه صدری بدخیم بوده و در صورت شک به وجود توده در جدار قفسه صدری بایستی اقدامات مقتضی جهت درمان به موقع و خروج توده انجام داد. یافته‌ها: از 112 مورد تومور بررسی شده 46 مورد(1/41٪) خوش خیم و 66 مورد (9/58٪ ) بدخیم بود . از طرف دیگر 35 مورد از تومورهای مردان و 31 مورد از تومورهای زنان بدخیم بود (5/56٪ در مقابل 62٪). در عین حال، آزمون مجذور کای نشان داد توزیع فراوانی نوع تومور در دو جنس تفاوت معنی‌داری ندارد(55/0=p). شایعترین علامت در مبتلایان به تومورهای خوش‌خیم (3/28٪) درد قفسه سینه و همچنین (3/28٪) توده برجسته و در مبتلایان به تومورهای بدخیم 19 نفر (8/28٪) درد قفسه سینه بود. شایعترین نوع بافت شناسی در تومورهای خوش خیم 15 مورد (6/32٪) التهابی و در تومورهای بدخیم 12 مورد (2/18٪) استئوسارکوم بود.

Title: تاثیر عصاره هیدرو- الکلی دانه خرنوب(Ceratonia siliqua L.) بر فاکتورهای عملکردی کلیوی و الکترولیت‌های سرم در موش صحرایی نر دیابتی

http://yafte.lums.ac.ir/article-1-444-fa.html

دیابت‌ملیتوس مهمترین بیماری متابولیک در انسان است‌که منجر به هیپرگلیسمی و هیپرلیپید‌می می‌شود. هیپرگلیسمی می‌تواند موجب آسیب‌کلیوی گردد.در این تحقیق تأثیرعصاره‌هیدرو-الکلی دانه خرنوب بر کراتی‌نین، اسید اوریک، نیتروژن‌اوره، سدیم، پتاسیم وکلسیم در موش‌صحرایی‌دیابتی‌نر مورد بررسی قرار گرفت. بحث و نتیجه‌گیری: بر اساس نتایج حاصل از این تحقیق عصاره هیدرو-الکلی دانه خرنوب می تواند موجب کاهش مشکلات کلیوی ناشی از دیابت شود. یافته‌ها: میانگین غلظت‌سرمی‌کراتی‌نین،نیتروژن‌اوره درگروه‌تجربی1و میزان پتاسیم در گروه‌های تجربی1و3کاهش معنی‌داری نسبت به گروه‌کنترل دیابتی نشان داد.در گروه تجربی1غلظت اسید اوریک و در گروه‌های تجربی1و3 غلظت سدیم افزایش معنی‌داری را نسبت به گروه‌کنترل‌دیابتی نشان داد.اختلاف معنی‌داری درغلظت کلسیم درهیچ‌کدام از گروه‌ها مشاهده نشد. مواد و روش‌ها: 56سرموش صحرایی‌نر‌بالغ ‌از نژاد ویستار، ‌با وزن تقریبی220-210گرم به‌طور تصادفی به هفت‌گروه‌هشت‌تایی تقسیم شد:گروه‌کنترل بدون هیچگونه تیماردارویی،گروه‌شاهدکه‌روزانه فقط آب‌مقطردریافت نمود، گروه‌کنترل تیمار شده باعصاره‌هیدرو-الکلی دانه‌خرنوب mg/kg)600)،گروه‌کنترل‌دیابتی که فقط استرپتوزتوسین دریافت کرد وگروه‌های تجربی 1و2و3 که یک هفته بعد از دیابتی‌شدن به‌ترتیب روزانه مقادیر150و300و600 میلی گرم به ازای کیلوگرم وزن، عصاره‌هیدرو-الکلی دانه‌خرنوب رابه صورت خوراکی و به‌مدت‌ 16روز دریافت کردند.در پایان دوره آزمایش میزان‌کراتی‌نین،اسیداوریک،نیتروژن‌اوره والکترولیت‌ها شامل سدیم،پتاسیم‌وکلسیم اندازه‌گیری‌شد. نتایج با استفاده از تست‌آماری ANOVAونرم‌افزارSPSS-18 تجزیه و تحلیل گردید و سطح 05/0p≤معنی‌دار در نظر گرفته شد.

Title: ارزیابی حساسیت سه پرایمر مختلف با استفاده از روش RFLP -PCR برای تمایز مولکولی مایکوباکتریوم‌های آتیپیک

http://yafte.lums.ac.ir/article-1-445-fa.html

با افزایش عفونت های ناشی از مایکوباکتریوم های آتیپیک (Non- tuberculosis mycobacterium NTM) در سال های اخیر، تشخیص سریع این دسته از اهمیت ویژه ای برخوردار است. از آنجایی که روش‌های فنوتیپی وقت‌گیر و پرهزینه هستند، امروزه از روش های مولکولی به طور وسیعی برای شناسایی و تمایز گونه های مختلف مایکوباکتریوم ها استفاده می‌شود. هدف ازاین مطالعه، تمایز مولکولی مایکوباکتریوم های آتیپیک با استفاده از سه پرایمر TB و 16S-23S rRNA gene spacer و hsp65 در روش RFLP -PCR و ارزیابی حساسیت این پرایمرها است. مواد و روش‌ها: بررسی روی 48 نمونه مایکوباکتریوم آتیپیک جدا شده از بیماران مبتلا به سل ریوی که توسط تست‌های فنوتیپی شناسایی شده بودند، صورت گرفت. حساسیت دارویی با روش نسبی (Proportional method) انجام و قطعاتی از ژنهای hsp65 ،16S-23S rRNA gene spacer توسط PCR تکثیریافت. سپس قطعات تکثیر یافته توسط آنزیم‌های AVaII، HpaII، HphI, HaeIII، BsteII هضم و الگوی بدست آمده روی آگارز 2% بررسی شد. بحث و نتیجه‌گیری: روش hsp65 PCR- RFLP از حساسیت و دقت بالایی برای تمایز گونه های مختلف مایکوباکتریوم‌های غیر‌توبرکلوزیس برخوردار است. یافته‌ها: از مجموع 48 نمونه، 8 نمونه (6/16%) MDR (مقاوم به چند دارو)، 4 نمونه ( 3/8%) حساس و 36 نمونه (75%) غیرMDR (مقاوم به یک دارو) بود. 13 نمونه (27%) تند رشد و سایر نمونه ها ( 73%) کند رشد بود. سرعت تشخیص پرایمر hsp65 در روش PCR- RFLP، در بسیاری از گونه ها بالاتر از بقیه پرایمر ها گزارش شد.

Title: تشخیص و درمان آنتی اسپرم آنتی‌بادی

http://yafte.lums.ac.ir/article-1-446-fa.html

ناباروری ایمونولوژیک ناشی از آنتی‌ اسپرم آنتی بادی‌ (ASA) یکی از علل مهم ناباروری در انسان‌ها است. شیوع ASA در زوج‌های نابارور 36-9 درصد گزارش شده است. شیوع ASA در مردان زوج‌های نابارور 21-8 درصد و در زنان زوج‌های نابارور 23-6 درصد می‌باشد.چندین روش برای تعیین ASA موجود هستند. در گذشته، اهمیت کلینیکی ASA به دلیل این واقعیت که روش استانداردی جهت تعیین ASA موجود نبود، مخفی مانده بود. اگرچه این موضوع باید روشن گردد که آیا هر آنتی بادی متصل به یک آنتی ژن شناسایی شده بر سطح اسپرم، عملکرد اسپرم را نیز تحت تاثیر قرار می‌دهد. روش‌های متعددی برای درمان ناباروری ایمونولوژیک گزارش شده‌اند. بیشتر تکنیک‌های موجود عوارض جانبی دارند، تهاجمی و پرهزینه هستند، کارایی پایینی دارند یا نتایج مغایری داشته‌اند. این مقاله مروری به افزایش دانش ما در مورد روش های تشخیص و درمان ASA کمک می کند.

Title: تظاهرات سندرم آلگرو (Allgrove Syndrome) بدنبال اسهال و استفراغ

http://yafte.lums.ac.ir/article-1-447-fa.html

سندرم آلگرو (Allgrove Syndrome) ،سندرم تریپل آ یا نقص فامیلی گلوکوکورتیکوئید یک نقص ژنتیکی نادر با توارث اتوزوم مغلوب است و اولین بار درسال1978 توسط الگرو و همکارانش گزارش گردید. سندرم آلگرو با فقدان اشک (آلاکریما )، نقص ایزوله گلوکوکورتیکوئید و آشالازی قسمت تحتانی مری شناسایی می شود. یافته‌های عصبی و پوستی نیز درتعدادی از بیماران دیده می‌شود. افزایش رنگدانه پوستی و فقدان اشک از یافته‌های کلیدی در تشخیص سندرم آلگرو است. در این مقاله دختر 2 ساله‌ای که بدنبال ابتلا به اسهال و استفراغ دچار افزایش رنگدانه پوست شد،گزارش می‌شود.
